# Current practices of psychoeducation interventions with persons with bipolar disorders: a literature review

**DOI:** 10.3389/fpsyt.2023.1320654

**Published:** 2024-01-05

**Authors:** Vanessa Levrat, Sophie Favre, Hélène Richard-Lepouriel

**Affiliations:** ^1^Mood Disorder and Anxiety Unit, Psychiatric Specialties Service, Geneva University Hospital, Geneva, Switzerland; ^2^Department of Psychiatry, University of Geneva, Geneva, Switzerland

**Keywords:** psychoeducation, bipolar disorder, psychosocial intervention, quality of life, mindfulness, group therapy

## Abstract

This review sought to summarize the literature on current practices and forms of psychoeducation in the management of patients with bipolar disorder (BD), including only randomized controlled trials to ensure the best level of evidence. An extensive review of the available literature was conducted using PubMed/MEDLINE, Embase, and PsychInfo databases from inception to April 28th, 2022. The search yielded 381 studies. Seventy articles were included after removing duplicates and applying the inclusion/exclusion criteria. A best-evidence synthesis was used to identify the key results of each study and summarize the outcomes. Eleven descriptive categories were made. They encompass different forms of psychoeducation compared or combined with other psychosocial interventions, varying in setting (individual or group), with or without family members, structured or unstructured, mediated or not by digital tools (smartphone, internet). Globally, these studies show that psychoeducation is important in the treatment of BD, as it leads to a decrease in relapses, mood episodes, hospitalizations, and improved functioning or quality of life. Some studies also showed the benefits of psychoeducation on the patient’s level of knowledge of pharmacological treatment and the disorder or compliance with medication, as well as reduced self-stigma. The limitations of this review are linked to the selection of only RCTs and the reliance on their post-hoc analyses. This review confirms the benefit of psychoeducation and psychosocial interventions on the evolution of BD (in different outcomes, including quality of life, relapse, and rehospitalization rates, for example). More recent interventions, such as mindfulness or online psychoeducation, represent an interesting option but more evidence is needed.

## 1 Introduction

Bipolar disorder (BD) is a mental illness, characterized by severe mood swings, impacting 1 to 6% of the population when the wider range of the bipolar spectrum is considered (1–4). In their review, Clemente et al. (5), showed that the pooled lifetime prevalence of BD-I was 1.06% (95% confidence interval [95%CI] 0.81–1.31) and that of BD-II was 1.57% (95%CI 1.15–1.99). Burdick et al. (6) found high rates of functional impairment (ranging from 41 to 75%) in BD. Functional impairment in BD is characterized as persistent and common and can be influenced by different key factors (such as depressive symptoms, lower levels of education, a greater number of prior mood episodes, comorbid substance use disorder, and the number of psychotropic medications).

Despite effective mood-stabilizing treatment (7), the relapse rate of patients was about 40% after 1 year, 60% after 2 years, and 73% after 5 years (8). Guidelines are updated and new treatments become available. Goldberg et al. (9) stressed the importance of collaboration between clinicians, patients, and their families to foster decision-making and develop individualized treatment. Drug treatment and psychosocial intervention is necessary to ensure the best possible clinical stability for patients living with BD (10). To date, different psychosocial interventions have been designed as adjunct strategies to pharmacotherapy in treating BD, and their effectiveness has been assessed. These interventions may be conducted with individuals or groups. They mainly rely on psychoeducation (PE), an evidence-based intervention for patients and their families that provides information about the illness (definition and cause, symptoms, pharmacological treatment, early detection of mood episodes, rhythm of life and stress management, and problem-solving) (11). Life Goals Program (LGP) was developed in the United States by Bauer et McBride (12, 13). It is a two-phase program. Phase I consists of five highly structured PE sessions focused on developing disorder management skills, such as identifying warning signs of relapse and developing effective coping strategies. Phase II focuses on identifying realistic personal goals in the social or professional spheres affected by the illness and developing strategies to achieve these goals with concrete and quantifiable means. Frank et al. (14) developed Interpersonal and Social Rhythm Therapy (IPSRT). This psychosocial intervention is specifically adapted for BD. It combines PE and cognitive behavioral therapy (CBT) elements, particularly in interpersonal relationships, with more specific elements focused on regulating biological rhythms. The concept is based on the hypothesis that an increase in the regularity of patients’ daily life (sleep/wake cycles, mealtimes, rest, and activity times) is associated with reduced chronobiological desynchronization. IPSRT is structured according to the different stages of the disorder. Functional remediation (FR) (15) is a specific program for BD patients with cognitive deficits during euthymic periods. Approximately 30% of patients with BD show cognitive deficits during the euthymic period, which can be assessed by neuropsychological tests (16). Approximately two-thirds present subjective cognitive complaints (17) that impact quality of life (QoL). FR therapy combines cognitive remediation and PE approaches. It aims to improve neurocognitive deficits to achieve functional recovery and improve clinical stability and QoL.

CBT programs are structured around an initial psycho-educational phase, an important part of the program, a second phase focused on specific CBT techniques (self-observation, various cognitive-behavioral strategies), and a third phase of consolidation (18–22). Mindfulness is a practice that involves focusing on the present moment with openness and acceptance. Several Mindfulness-Based Interventions (MBI) exist, such as Mindfulness-Based Cognitive Therapy (MBCT) which is a program developed to prevent depressive relapses (23). This approach combines cognitive psychotherapy with elements of meditation practice and PE. These groups include people who have already experienced mood disorders (unipolar or bipolar) and who are in remission or with mild symptomatology.

PE was also developed with families of persons living with BD. Miklowitz developed family focused therapy (FFT) in the late 90s. It combines family PE sessions with teaching strategies for improving communication and solving problems related to the disorder or the family environment. The 21 sessions are adapted to each stage of the disorder (acute, maintenance, and consolidation phases). Other interventions have been developed (e.g., collaborative care, psychosocial rehabilitation, online psychoeducation). In this literature review, the focus is on the current forms of psychosocial interventions in managing BD.

Soares-Weiser et al. (24) conducted a review to determine the clinical effectiveness and cost-effectiveness of pharmacological and/or psychosocial interventions in managing BD. They showed that some medications (lithium, valproate, lamotrigine, and olanzapine) were effective as maintenance therapy for the prevention of relapse in this condition and that some psychosocial interventions (CBT, PE, and family therapy) might be beneficial as adjuncts to pharmacological maintenance treatments. The same year, Weber Rouget et al. (25) reviewed the efficacy of PE and recommended PE as part of the integrated treatment of BD, in individual or group settings. In their review, Reinares et al. (26) showed that adjunctive psychological treatments (such as CBT, PE, interpersonal rhythm and social rhythm therapy, cognitive and functional remediation, and family intervention) could improve BD outcomes. They recommend interventions to be introduced as soon as possible to improve prognosis, and that they should be adjusted and personalized. A recent systematic review (27) focused on the role of psychoeducation in BD in patients and their families. They searched Medline, Scopus, and Lilacs databases with the terms “bipolar disorder” and “psychoeducation” and showed that psychoeducation interventions applied to BD patients and their relatives were effective in reducing the frequency of new mood episodes, as well as the number of hospital admissions and length of stay.

The aim of this literature review was to investigate the different practices and forms of PE with persons with BD and/or their relatives. The goal was to understand how PE was used by itself and identify its use in combination with other psychosocial interventions, and how these interventions may develop.

## 2 Methods

The Preferred Reporting Items for Systematic Reviews and Meta-Analyses (*The Patient, Intervention, Comparison, and Outcome*) is an evidence-based set of items for reporting in systematic reviews and meta-analyses. Even though this is a literature review, we used the pertinent items of the PRISMA 2020 Checklist (28) to guide the methodology of this review (eligibility criteria, information sources, search strategy, selection process, data collection process, data items, effect measures, synthesis methods).

### 2.1 Search strategy

A comprehensive systematic computerized search was performed on three academic databases –PubMed, Embase, and PsycInfo – from inception until April 28th, 2022. Search syntax was compiled using the related entry terms from MESH and relevant keywords. Boolean operators (AND, OR) were used to compile the search syntax. The search strategy used included: (bipolar disorder) AND (psychoeducation) AND ((randomized clinical trial) OR (randomized controlled trial) OR (clinical trial)).

### 2.2 Eligibility criteria

Relevant articles were included on the following criteria: Randomized controlled trial (RCT) peer-reviewed studies involving psychoeducation for adult patients diagnosed with BD. Studies with participants with serious mental illness (SMI) were also included when BD was represented.

Articles were excluded on the following criteria: studies with participants under 18 years old or assessing psychoeducation only in families. Articles in languages other than English or French, conference abstracts, study protocols, and reviews were also excluded.

The Patient, Intervention, Comparison, and Outcome (PIC0) model (29) was used as a framework to build the review question (Table 1).

**Table 1:** The inclusion and exclusion criteria.

PICO	Inclusion	Exclusion
P: BD-I or BD-II and SMI I: psychoeducation (group, individual, family) C: combined interventions O: mood, hospitalization rates, quality of life	RCT peer-review studies Adults PubMed, Embase, and PsycInfo English and French language No time restriction	Not RCT >18 years old Grey literature Other languages Conference abstracts, study protocols, reviews

BD, bipolar disorder; C, comparison; I, intervention; O, outcome, P, population, RCT, randomized controlled trial; SMI, serious mental illness.

### 2.3 Article selection

Two authors (VL and SF) independently screened titles and abstracts for article eligibility following the inclusion/exclusion criteria. A template was developed to record the decision made by each reviewer as to whether the paper should be included or excluded or if the decision was uncertain. These decisions were compared, and disagreements were resolved through discussion until consensus was achieved.

## 3 Results

### 3.1 Description of the included studies

**Figure 1:**
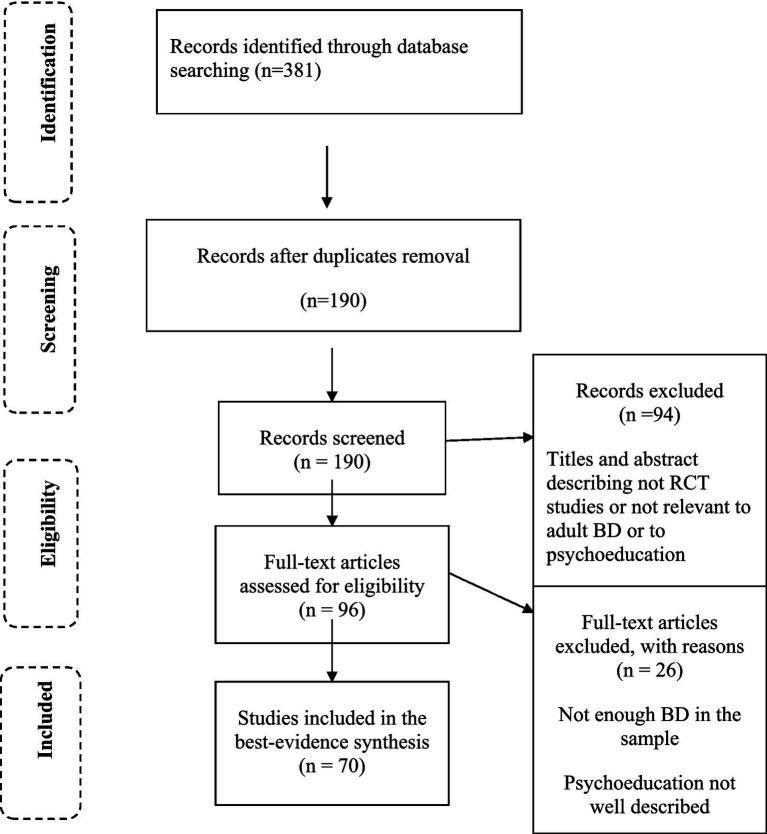
Flow chart of the review process.

The search yielded 381 studies. Seventy articles were included after removing duplicates and applying the inclusion/exclusion criteria described in Figure 1 [PRISMA flowchart (30)].

### 3.2 Data extraction

The categories were chosen to summarize the main features of the included studies with the focus of this review. The synthesis encompassed the characteristics of the populations, interventions, and outcomes. The “population” category describes whether BD-I and/or II were included in the study, the “number of patients” category shows the magnitude of the study sample, the “groups” category describes the experimental group (EG), and the control group (CG) of the RCT study, the “treatment” category presents a more specific description of both EG and CG, the “PE: length and frequency” category details the number of sessions, the “follow-up” category refers to the duration of the monitoring after the intervention, and the “results” category reports on the findings of the studies. These categories enabled a comparison of the different RCT studies that globally showed the diversity of the practices with PE. Some studies were grouped as they shared commonalities, such as working with a specific PE model, combining interventions, or comparing interventions.

Supplementary Table S1 presents PE models and other psychosocial approaches classified the selected studies. For all articles, data concerning the first author, year of publication, number of patients included, type of psychiatric pathology targeted, description of the EG (type of PE/care received, duration, frequency, associated tools) and of the CG, as well as relevant primary and secondary outcomes were collected. Other more specific data were extracted, depending on the article’s content.

### 3.3 Data synthesis

Theme extraction was done by identifying key concepts and findings of the selected studies. The studies were organized into different categories. These categories enabled a comparison of the different RCT studies that globally showed the diversity of the practices with PE. Some studies were grouped as they shared commonalities, such as working with a specific PE model, combining interventions, or comparing interventions.

Overall, there were 18 studies related to group PE and the model of Colom and Vieta, and eight with the Bauer and McBride model. In the psychotherapeutic and psychosocial approaches category, five articles were related to CBT interventions [two of which also dealt with FFT and with interpersonal and social rhythm therapy (IPSRT)], five with FFT (two of which were common with CBT and IPSRT) and three with therapies including the family, which were less specific than FFT. Four articles dealt with IPSRT (two of which were common with CBT and FFT), and six studied FR’s effects. There were four articles in the rehabilitation programs and collaborative care category. Three articles did not specify a particular model, and two others addressed specific needs of patients with BD. Twelve articles described PE based totally or partially on digital models (mobile application, SMS reminders, internet). Other types of intervention were less represented, such as dialectic behavioral therapy (DBT) group skills training for BD and mindfulness-based cognitive therapy for BD (four studies).

#### 3.3.1 The Colom and Vieta model

The Colom and Vieta model of PE (11) is structured in 21 sessions of group intervention, aiming at improving illness awareness, treatment compliance, early detection of prodromal symptoms and recurrences, and lifestyle regularities. This model is designed as an adjunctive treatment in pharmacologically treated euthymic outpatients with BD-I and BD-II, to prevent the emergence of a new acute episode.

Colom et al. conducted two studies in 2003. They compared a structured PE program to a group receiving treatment as usual (TAU) in fully compliant patients with BD-I (31). In the first study, PE participants had better outcomes than those with TAU on different measures (relapse rates, number of recurrences and depressive episodes, number and length of hospitalization, increased time to depressive, manic, hypomanic, and mixed recurrences) at 2 years follow-up. In the second study (32) patients with BD type 1 were included. The results at two-year follow-up were similar to those of the first study and no significant difference related to medication adherence was found. These results were maintained at a five-year follow-up (33, 34). PE was also useful for bipolar patients with personality disorder (35).

Based on the Colom et al. study (31), Scott et al. (36) examined the long-term (5-year) cost–benefit effect of PE groups with BD-I and BD-II. PE at follow-up was less costly and more effective compared to TAU. The share of hospital care in total costs was significantly lower for psycho-educated patients, as well as the estimated average cost of emergency visits. Javadpour et al. (37) showed that at 18 months follow-up, participants with PE had significant improvement in medication compliance, QoL, and a decrease in the number of relapses and hospital admissions compared to TAU.

Three Brazilian studies were conducted with young patients (18–29 years) with BD. The first one (38) focused on the effect of PE (six individual sessions) on biological rhythms and depressive symptoms in BD-II patients compared to TAU. Both groups showed remission of depressive symptoms and no reduced manic symptoms. PE had no significant effect on regulating biological rhythms (sleep, activity, social rhythm, eating patterns). The second study (39) investigated the impact of this PE on QoL and there was no significant difference between the groups on QoL. The third study (40) focused on the evolution of symptomatology and the regulation of biological rhythms (12 months follow-up). The evolution of depressive, manic, and anxiety symptomatology or the regulation of biological rhythms did not differ. However, there was a favorable trend for PE participants.

Kallestad et al. (41) compared the long-term (8 years) effect of a group PE based on Colom and Vieta’s models and Miller and Rollnick’s model (42) (motivational interviewing) to a short individual PE of three sessions. The persons with the first intervention (PE) had a significantly longer time to re-hospitalization, not due to the type of PE, but to other factors (substance use, number of previous hospitalizations, younger age, and BD-II). This trend continued at the end of the follow-up. Recently, Harmanci et al. (43) showed that PE and motivational interviewing have positive effects on improvement in treatment adherence and functionality in persons with BD.

PE was effective on other outcomes, such as medication adherence (44), and relapse rate (12 months) (45) when compared to TAU. However, there was no difference between the groups regarding the evolution of depressive, hypo(manic) symptomatology, or treatment compliance between a group receiving PE and TAU combined with free discussion groups (46). Wiener et al. (47) studied the impact of PE on serum levels of g brain-derived neurotrophic factor (BDNF), nerve growth factor (NGF), and cell line-derived neurotrophic factor (GDNF) levels in young adults with BD. An increase in GDNF levels was noted for EG only, at the end of the PE’s sixth session. Serum levels of the other trophic factors studied did not change significantly pre-and post-intervention. There was a decreased severity of depressive symptomatology in PE and TAU, and no effect on (hypo)manic symptomatology.

Other studies compared a structured group-based PE, and the second an unstructured group-based PE based on peer support. The results showed a significantly higher rate of participation in structured PE (14 sessions vs. nine sessions) (48). The structured PE intervention had a net incremental cost with a small QALY (Quality-Adjusted Life Year) gain over the CG. However, the authors noted high uncertainty in the data and results (49).

Chen et al. (50) compared two different interventions: the first group (EG) received a group-based, structured PE adapted from Colom and Vieta’s textbook model for the Chinese population, and the second (CG) received the same textbook but with free-flowing, unstructured focus groups. At 1 year, the difference between the groups for the number of manic relapses was significant and in favor of the CG as well as the number of re-hospitalizations. A significant improvement between the beginning and the end of the follow-up was present for the EG on depressive, (hypo)manic symptomatology and global functioning. Medication compliance was similar in both groups.

#### 3.3.2 Psychoeducation programs using the Bauer and McBride model with patients with BD

The Bauer and McBride model of PE (12) was designed to enhance patient self-management skills. It is structured into eight sessions aiming at improving knowledge of BD, mania, and depression, as well as treatment for BD.

Bauer et al. (51, 52) evaluated the effectiveness of their PE program (LGP) versus TAU and showed a significant reduction in duration in manic episodes, but not in depressive episodes. PE benefited overall social functioning, including work and parenthood, but not leisure or couple sphere. QoL was improved only for the mental domain. Satisfaction with treatment was better at 6 months for PE. There was no significant difference regarding the doses of psychotropic drugs taken by the participants. At 3 years of follow-up, the PE group had better concordance rates for antimanic treatments than TAU (53). Simon et al. (54) also showed a significant reduction in the mean level of manic symptoms and manic symptomatology duration in the PE program compared to TAU, and no significant effect on depressive symptoms. The authors also compared the cost–benefit ratio, showing a modest increase in the cost of PE-related care compared to the benefits obtained. Parikh et al. (55) showed that PE is less expensive than CTB. They compared the relative effectiveness of a brief PE (six sessions) and a CBT intervention (20 sessions). The reduction of symptom burden and the likelihood of relapse was similar for both treatments. Sajatovic et al. (56) investigated the impact of LGP compared to TAU on beliefs and attitudes towards drug treatments. They showed that attitude towards pharmacological treatment was improved at three and 6 months in the LGP group compared to TAU, but with a non-sustainable effect at 12 months.

#### 3.3.3 Psychoeducation programs compared to or combined with CBT interventions

A pilot study (57) evaluated the efficacy of adding a CBT (13 individual sessions) to a standard brief PE (seven individual sessions). Participants who received CBT in addition to PE experienced fewer days of depressed mood over 1 year and had less antidepressant increases compared to those with PE only. González-Isasi et al. (58) evaluated the efficacy of a combined treatment (pharmacological + psychoeducational and cognitive-behavioral therapy) as compared with standard pharmacological treatment in patients with refractory BD with repeated measures at separate times. Combined treatment yielded a lower rate of hospitalization (at 12 months), and lower rates of depression and anxiety (at six and 12 months). These results were maintained at five-year follow-up (59).

#### 3.3.4 Psychoeducation programs combined with DBT and mindfulness interventions, with MBCT, and with specific yoga technics

Van Dijk et al. (60) explored the combination of standard PE with DBT and mindfulness-based techniques. At the end of the study (12 weeks), the participants showed a reduction in depressive symptoms compared to CG. In addition, the Mindfulness Efficacy Scale (MSES) showed an improvement for both groups, although it was higher for the EG group. Moreover, the participants in the EG group had fewer psychiatric emergency room visits in the 6 months following treatment. Dios et al. (61) compared TAU with PE and with MBCT. At 6 months follow-up, there was a similar decline in the severity of depressive symptoms in the three arms. There was no significant difference on other measures (anxiety, hypo/mania symptoms, functional functioning). Ravindran et al. (62) assessed alternative combination of PE and a breathing-focused yoga and showed a similar decline in depressive symptoms and that the intervention order made no difference. Valls et al. (63) compared TAU with an integrative approach (PE, mindfulness training, and functional remediation) that showed improvement in psychosocial functioning and residual depressive symptoms in favor of the patients who received the integrative approach versus the TAU group.

#### 3.3.5 Psychoeducation programs with family members of patients with BD

Miklowitz et al. (64) compared Family-Focused Therapy FFT (21 sessions) to a two-session (family education) and follow up crisis management (CM), over a one-year period. The FFT participants had fewer relapses and longer delays before relapses than the CM participants. Greater improvement in depressive symptoms was also observed in FFT. At 2 years of follow-up (65), there were fewer relapses and longer delays before relapses, as well as a greater reduction in depressive and manic symptomatology and better compliance with medication in FFT. Rea et al. (66) compared FFT to an individual PE approach. There was no difference between the two groups regarding the probability of mood relapse, at one-year follow-up. At 2 years follow-up, there were less relapse and hospitalizations in the FFT group. Both groups showed good pharmacological compliance. A comparison of three types of interventions (FFT, CBT and Interpersonal and Rhythm Therapy (IPSRT)) to CG showed that the groups of patients receiving one of the three models of psychotherapy had a significantly higher rate of remission at 12 months and shorter time to remission and were also 1.58 times more likely to be clinically stable during the study period compared to the CG group. Moreover, these three types of psychotherapy improved social and relational functioning and life satisfaction after 9 months follow-up. However, there were no significant differences between the three types of intensive psychotherapy (67, 68).

Three studies assessed other interventions with family members. Clarkin et al. (69) compared the impact of adding marital intervention to medication management. PE for couples was associated with improved medication adherence and overall functioning, but the intervention did not affect patients’ symptomatology. In a similar model of PE involving patient and spouse (EG), D’Souza et al. (70) showed that there were less risk of relapse and a longer mean time to relapse in the EG compared to the CG. At the end of the study, the EG had lower scores related to (hypo)mania and better compliance with medication. There was no difference between the groups for depressive symptoms.

Pakpour et al. (71) described an intervention combining PE for patients and their families and motivational interviewing. Motivational interviewing (42) aimed at decreasing resistance and ambivalence to taking treatment. Medication adherence was improved at 6 months, as well as of clinical symptoms, measures of intention, beliefs about medication, perceived control, automaticity, and planning around taking mood stabilizers, but not QoL.

#### 3.3.6 Psychoeducation programs compared to interpersonal and rhythm therapy (IPSRT)

Frank et al. (14) compared IPSRT with Intensive Clinical Management (ICM). The results showed that the groups receiving IPSRT in the acute phase had more time without mood relapse and a better regularity in social rhythms at the end of the acute phase of treatment. However, no difference was found in the time to reach clinical stabilization. A more rapid improvement in occupational functioning was also present for the IPSRT groups. The results were the same at 2 years (72).

#### 3.3.7 Psychoeducation programs compared to functional remediation

After comparing PE, FR, and TAU (medication), Torrent et al. (73) reported that FR significantly improved functioning compared to the TAU group. However, there were no significant differences between groups regarding clinical or neurocognitive variables at the end of the study. Solé et al. (74) isolated a subgroup of subjects with BD-I and BD-II from an RCT conducted by Torrent et al. (73). The results favored FR over PE and TAU to improve functioning. No differences were observed between the groups in the evolution of subclinical manic symptoms. Sanchez-Moreno et al. (75) conducted a sub-analysis of the study by Torrent et al. (73) involving a subsample of patients with subsyndromic symptomatology to assess the effect of PE at 12 months. The RF group significantly improved psychosocial functioning compared to the TAU and PE groups. No significant changes in mood symptoms were observed in any of the three groups. Bonnin et al. (76) isolated a subgroup from Torrent et al.’s (70) study sample with neurocognitive performance within two standard deviations of the mean. The FR group, compared to the TAU group, showed better performance in one aspect of the neurocognitive testing (free recall (verbal memory)). However, the outcome was not significantly different from the PE group. Functioning was significantly improved in the FR group compared to the other two groups. Bonnin et al. (77) investigated whether the effects of FR persisted at 1 year and 6 months follow-up. Changes in the FAST scale score revealed a significant improvement in functioning in the FR group from baseline to 1 year compared to the other two groups. The secondary results concerning neuropsychological changes showed contrasting results, with a significant improvement in favor of the FR group concerning verbal memory. However, no difference was observed between the groups concerning other neurocognitive variables (i.e., executive scores, processing speed, working memory, and attention). Sachs et al. (78) compared cognitive PE with TAU. Compared to healthy controls, patients with BD show lower executive function, verbal learning, and fluency performance. Cognitive PE and attention predict occupational function. Verbal memory recall is a predictor of symptom severity.

#### 3.3.8 Psychoeducation programs combined with rehabilitation programs and collaborative care

Van der Voort et al. (79) compared the effectiveness of collaborative care (CC), which integrates PE and elements of problem-solving, to TAU (CG). At 12-month follow-up, the CC group showed a significant reduction in the number of months spent with depressive symptoms at six and 12 months of follow-up and a reduction in the severity of depressive symptoms at 12 months. The intervention did not affect manic symptomatology or medication compliance. Dalum et al. (80) compared a group receiving TAU with an Illness Management and Recovery (IMR) program, combining PE and care concepts belonging to the psychosocial domain. The IMR is not specific to BD. It was developed to help patients improve their knowledge and management of their psychological and long-term illness and to achieve clinical and personal recovery. No significant differences were found between the groups at 9 months (duration of the program) regarding self-management of the disorder, hope, remission, or satisfaction with treatment. Owing to the lack of conclusive results for the IMR program, a second study was added to that published in 2019 (81). This study focused on the outcomes at a one-year follow-up. Nevertheless, no significant differences were found between the EG and CG in global functioning, symptomatology, number of hospitalizations, emergency department visits, or outpatient care utilization. Shon et al. (82) explored the impact of a non-BD-specific PE program combining PE with rehabilitation concepts, partly based on Bandura’s (83) self-efficacy method. The effects of the EG were quite positive, with a greater improvement in self-efficacy and medication compliance and a greater reduction in relapse symptom scores.

#### 3.3.9 Psychoeducation programs and unspecified theoretical model

Luciano et al. (84) compared a classic PE group with a psychosocial lifestyle group and a brief psychoeducational group in terms of their impact on body mass index in patients with SMI. This intervention effectively improved the physical health of patients with SMI. So et al. (85) compared a specific PE (Life Goals Program (LGP)) to a waiting list. Significant improvements in knowledge about illness and anxiety levels in medication adherence were present at the six-month follow-up in the LGP group. The two RCTs conducted in Turkey did not specify the PE models used. Many elements appear common to Colom ‘sand Vieta’s PE; however, this has not been explicitly stated. Eker et al. (86) compared the effectiveness of a six-week PE program with that of CG on medication adherence. At the end of the PE program, there was a significant difference in medication adherence between the EG and the CG. Çuhadar et al. (87) explored the effect of PE on self-stigma. At the end of the follow-up period, the EG showed a significant reduction in the total score of the Internalized Stigma of Mental Illness Scale (ISMI) and sub-items on the level of alienation, stereotype endorsement, perceived discrimination, and social withdrawal. Recently, Latifian et al. (88) showed that PE can be useful to reduce internalized stigma in family members of persons with BD and to increase their positive attitudes towards psychological disorders.

#### 3.3.10 Psychoeducation programs with patients with specific needs

Harvey et al. (89) applied a CBT model to patients with BD-I and insomnia and compared it with non-specific PE. During 6 months of follow-up, the CBT group showed better outcomes, with fewer days spent in an acute episode, fewer (hypo)manic relapses, a slightly lower overall relapse rate, and reduced insomnia severity. A higher remission rate was observed after treatment but not at 6 months.

Sajatovic et al. (90) focused on an intervention called Targeted Training in Illness Management (TTIM) derived from LGP and developed it for patients with psychological pathology and diabetes. This group combined PE, problem identification, goal programming, and behavioral modeling. Greater improvements in depressive symptomatology, global clinical impression, and global functioning were observed in the TTIM than in the CG. No differences were observed between the groups regarding general health, psychological symptoms, or mean glycated hemoglobin levels.

#### 3.3.11 Psychoeducation programs based totally or partially on digital models

Smith et al. (91) compared an internet-accessible PE (“Beating Bipolar”) to TAU and found no significant differences between the two groups in QoL, patient functioning, insight, mood, and the number and severity of relapse at 10 months follow-up. Proudfoot et al. (92) compared an eight-week online psychoeducation program, a PE program with online peer support, and an attentional control condition. There was no significant difference among the three groups, except for a slight difference between the two groups receiving PE in favor of the group with additional peer support for depressive symptomatology and functional impairment. Program adherence was also significantly higher.

Barnes et al. (93) compared the effectiveness of a computerized program called “Health-Steps for Bipolar Disorder” combining PE and optional CBT sessions with a CG receiving only links to healthy lifestyle sites. At the 12-month follow-up, the groups had no differences regarding the time to recurrence.

Lauder et al. (94) compared a PE program (Mood Swings (MS)) combined with a patient-to-patient discussion forum with MS-PLUS, a more interactive version of MS combined with a discussion forum that included CBT tools. Both groups showed reduced mood symptoms and improved functioning, quality of life, and medication compliance during follow-up. Greater within-group improvements in symptoms and functioning for depression and mania, QoL, and social support were observed in the MS-PLUS and mania scores. There were no differences in the relapse rates between the groups. Different levels of Mood Swings 2.0, one with interactive CBT tools (EG2) and one without (EG1), were compared with a CG supported by peer-supported forum discussions (95). The results showed a significant positive impact on depression (EG1 vs. CG). No significant differences were found between the QoL, compliance, and mental health functioning groups.

Depp et al. (96) compared two groups receiving four sessions of PE, one supplemented with 10 weeks of personalized interactive smartphone monitoring and a CG performing simple self-observation (paper diary) mood monitoring. Smartphone tracking allowed the participants to generate personalized self-management strategies according to their mood. A high satisfaction rate was observed for both groups. Both the PE groups showed a greater reduction in depressive symptoms at six and 12 weeks than the CG. However, these effects were not observed after 24 weeks. The mania scores and functional impairment did not differ between groups.

Moore et al. (97) studied PE in patients with BD and HIV, specifically, its effect on adherence to antiretroviral (ARVs) and psychotropic drugs. After a short PE targeted at medication adherence, the EG (individualized Texting for Adherence Building (iTAB)) received in parallel personalized SMS reminders with reinforcement/encouragement messages and reminders to take ARVs and psychotropic drugs with mood assessment. The average medication adherence was high and comparable between the groups. Participants in the iTAB EG took ARVs closer to the scheduled time than those in the CG; however, no significant difference was found between the groups in terms of the time of psychotropic medication.

Bilderbeck et al. (98) compared two methods of PE administration (supplemented by five individual sessions with a therapist or a second self-administered session only). Both measured weekly online mood self-observations. No difference was observed between the groups at 12 months in the evolution of depressive symptomatology or the relapse and hospitalization rates. The group benefiting from additional sessions with a therapist had better knowledge of BD than the CG (3 months) and a greater proportion of weeks of clinical stability over 12 months.

Dupuis Maurin et al. (99) compared PE followed by BIPOLIFE, with PE followed by TAU. BIPOLIFE provides information on BD and features an avatar with BD that must react to everyday situations. The objective was to learn how to better regulate an avatar’s mood and energy level by making daily life choices (such as activities, sleep, taking or not taking substances, taking treatments, and contacting caregivers). The game ended when euthymia was achieved for three consecutive days. The connections to BIPOLIFE were relatively low, despite user satisfaction. There was no significant difference in medication adherence at 4 months or in the number of consultations with a psychiatrist (scheduled or emergency).

Petzold et al. (100) combined 6 weeks of face-to-face PE with self-observation and data reporting on a Chrono-Record (EG) computer program. Participants in the EG were asked to report the following data daily: mood, sleep, life events, weight, menstrual cycle, and medication. The CG consisted of participants who received only non-specific and unstructured self-observation groups and instructions. After 2 years of follow-up, the study showed no significant difference in the number of relapses. The time to first relapse (depressive or hypo(manic)), QoL, perceived involvement in care, self-efficacy expectations, and health locus of control did not differ significantly between the groups. Depp et al. (101) compared CBT2go (one individual CBT PE session), self-monitoring (SM) (PE session for all diagnoses), and TAU in patients with SMI. PE was mediated using a smartphone application (self-observation of symptoms). The modest but significant improvements in the BPRS were similar and present for both interventions compared with the CG.

Murray et al. (102) compared two online interventions for 10 BD episodes. Both were five-week coach-supported programs, with TAU continued. One was a brief online mindfulness-based intervention (ORBIT 2.0) that did not prove superior to PE control in improving QoL.

## 4 Limitations

Most authors of the included studies discussed the limitations of the RCTs. They mentioned for example, the small sample size, the need for longitudinal data, the un-generalizability of the results (due to the type of treatment as usual, gender distribution in the sample, and specific culture), reliance on self-reports only, the study rated non-blinded to treatment, difficulty to isolate which part of the treatment was most effective.

This review aimed to provide a landscape view of the current uses of PE. Due to the heterogeneity of the selected studies, a quantitative meta-analysis could not be conducted to yield the pooled data on psychoeducation in BD. A best-evidence synthesis was used to identify the key results of each study and summarize the outcomes. The limitations of this review are linked to the selection of only RCTs and the reliance on their post-hoc analyses. We also note that there have been few studies on certain models, such as CBT, in our selection, although they have been widely studied for BD using other designs. Heterogeneity in the included RCTs was related to the variability in the participants and the definitions of outcome, clinical remission, and follow-up period. The risk of bias was not assessed as the aim of this review was not related to efficacy, but to describing practices. For the same reason, indirect group comparison was not needed.

## 5 Discussion

The goal of this literature review was to summarize evidence from RCT studies of different interventions for the same condition (BD) and to summarize evidence from RCT studies of different interventions for the same condition (BD) where different outcomes were addressed to provide a broad picture of practices with PE. This literature review yielded studies on different types of PE, varying in setting (individual or group), sometimes with sessions with family members, structured or unstructured, specific, or not, carried out with one or more caregivers, and/or partially mediated by digital tools (smartphone, Internet). Most studies included euthymic patients with BD-I and BD-II or who were minimally symptomatic. Clarkin et al. (69), Simon et al. (54), Bauer et al. (51, 52), Kallestad et al. (41), Barnes et al. (93), Miklowitz (67, 68), and Van Dijk et al. (60) recruited patients in the more acute phase of the disease. Scott et al. (103) showed that patients who were still symptomatic and had experienced a high number of past episodes did not benefit from PE.

In their review, Reinares et al. (26) discussed the probable difference in needs and responses to treatment early in the illness from more chronicity or complexity, as well as new treatments and the adaptation of other therapeutic approaches. Staging models provide a framework to identify illness trajectories and optimize treatment. In their review, Muneer et al. (104) investigated the literature on staging models in BD. They cited Duffy’s four-stage integrative clinical staging model for BD (105), which describes the course of BD according to illness subtypes. Other staging models were also discussed with their merits and limitations. This review showed how the staging model helps formulate individually tailored treatment plans in patients with BD. Berk et al. (106) discussed the clinical utility of this framework. They present a stage progression model that is combined with specific interventions at different stages (from lifestyle to CBT, pharmacology, mood stabilizer and psychoeducation, psychosocial strategies, relapse prevention, combination of mood stabilizers, plus functional-cognitive remediation). The general proposition of the staging model is that early intervention is more effective and needs to be less complex than later intervention. Marchionatti et al. (107) reviewed studies to assess the impact of disorder progression in treating persons with BD and reported an overall loss of efficacy according to clinical progression. They suggested using clinical staging models for future studies to strengthen methodology in this research field. Reinares et al. (108) showed that lower age at diagnosis of BD, lower cyclothymic temperament, and gender (male), which was associated with BD-I and more previous mania, were associated with a better response to psychoeducation. They recommend that existing programs be adjusted to the characteristics of the patients involved. Recently, Ratheesh et al. (109) conducted a systematic review of the effectiveness of interventions in the early course of BD I or II and showed the importance of offering effective pharmacological and psychological interventions in the early course of BD.

PE often appears to be a component of a complex and varied psychosocial approach. However, identifying the therapeutic effects of PE alone is challenging (25, 110). CBT, as an adjunct to PE, provides benefits over PE alone in terms of the time spent on depressive symptoms (57). Harvey et al. (89) also reported clear benefits with fewer acute episodes, fewer (hypo)manic relapses, and benefits for insomnia but used very specific CBT targeted at sleep disorders. In contrast, Parikh et al. (55, 111) showed non-significant differences between PE (Bauer and McBride model) and CBT, notably for the probability of relapse, symptoms, and medication compliance. Only certain more specific characteristics, such as denial or blame, showed improvement after CBT. The studies conducted by Miklowitz et al. (67, 68) also showed its superiority over PE in terms of the stabilization of bipolar depression, improvement in relationship functioning, and life satisfaction. Nevertheless, these results were valid for the three types of intensive psychotherapy (IPSRT, CBT, and FFT), with no significant differences. A literature review conducted by Reinares et al. (26) highlighted contrasting results regarding CBT studies, indicating the need for more research to clarify the effectiveness of this intervention and the factors that predict treatment responses. However, CBT appears to be particularly useful in preventing depression. These results are consistent with other reviews on this topic, such as those recently conducted by Novick et al. (112) and Khazaal et al. (110). The latter study highlighted two points: CBT tends to reduce depressive symptoms, improve treatment adherence, and reduce the risk of manic or depressive relapse. However, CBT and psychoeducational treatments share the same model of illness. As mentioned above, it is difficult to distinguish the impact of one intervention from another.

A more detailed analysis reveals disparities depending on the model used. Studies based on the models of Colom and Vieta or Bauer and McBride mainly showed benefits in reducing the number of relapses (mainly manic) and lengthening the time between relapses and the number of hospitalizations. Few effects have been reported on acute symptoms, particularly depression and compliance with medication. Conversely, Rahmani et al. (44) reported better pharmacological compliance among women with BD-I. Chen et al. (50) highlighted improved depressive and manic symptomatology but only targeted patients with BD-I hospitalized for a manic episode. Sajatovic et al. (90) also found an improvement in depressive symptomatology in patients with psychiatric pathologies other than BD-I. Only three studies found no benefit of PE compared to TAU, including studies by De Azevedo Cardoso et al. (39, 40) and Gumus et al. (45). PE by Bauer and McBride and Colom and Vieta showed strong evidence of efficacy in maintenance treatment, that is, outside the acute phases. PE is reportedly most effective when performed in groups, as shown in the RCTs by Colom et al. (32–35), D’Souza et al. (70), and Kallestad et al. (41). These results are consistent with those reported by Novick et al. (112). Adding more techniques (DBT, mindfulness, yoga) seemed to provide remarkable results, especially in reducing depressive symptoms, improving affective control, and the feeling of self-efficacy of mindfulness. However, the limitations of this study do not allow us to generalize these results.

The same is true for the FFT. FFT reduces the risk of relapse and hospitalization (66) and improves depressive symptoms, but not manic symptoms (64), as well as the return to post-episode euthymia and patient compliance with medication (65). Despite these benefits, implementing FFT is relatively restrictive, as it requires many sessions over a long period (9 months). It is also necessary for therapists to be trained and qualified for this approach, making it relatively inaccessible and expensive.

More accessible and less specific, the participation of relatives in a few sessions was evaluated in three studies. These interventions showed benefits in medication adherence (69–71), a decrease in relapse rate, a longer time between relapses, and a decrease in manic symptoms (70). However, this type of PE was compared to a TAU; therefore, it is unclear which component of individual or closed-knit PE provides benefits. Larger PE based on rehabilitation programs, including various psychiatric pathologies (80, 81), showed no benefits compared to TAU. Only the study by Shon et al. (82) found benefits, particularly regarding pharmacological compliance and feelings of self-efficacy. However, the low percentage of patients with BD in this RCT (<30%) should be emphasized, which makes it impossible to rely on these results for our groups. Recently, Okazaki et al. (113) showed that medication attitudes acquired through psychoeducation and program satisfaction impacted positively long-term medication adherence and QoL.

The outcomes from digital interventions are relatively modest and mixed. Smith et al. (91) showed a small effect of these tools on psychological QoL. Gliddon et al. (95) and Depp et al. (96) reported a favorable effect on depressive symptomatology, but only in the short term for the second study. Lauder et al. (90) found a reduction in mood symptoms, improved functioning and QoL, and medication compliance, valid for both digital PE alone and supplemented by CBT tools, not allowing a clear distinction of the effectiveness of each element. Several authors have reported no benefits associated with these forms of psychoeducation (93, 100) despite reasonable satisfaction and acceptability rates for these digital programs (96, 99), the number of connections is often relatively low, limiting the reliability of the results (99). Moreover, reports on long-term medication adherence benefits are rare. A systematic review of psychoeducational smartphone applications by Nicholas et al. (114) highlighted other negative points, including that the content often does not comply with the guidelines. Apps also fail to provide vital information to help users assess their quality and lack references to the sources of information used and privacy policies. In their review, Soo et al. (115) emphasized the paucity of RCTs in Internet psychoeducation. Piras et al. (116) investigated the quality of information on the treatments of BD available on English and Italian websites. Their findings show that these websites were difficult to read and required education; limiting some users to benefit from this support.

This review confirms the benefits of PE and psychosocial interventions in the evolution of BD in almost all types of interventions studied. In their review, Rabelo et al. (27) also showed the positive effects of PE on both patients and family members. In this review, the focus was on PE and combined psychosocial interventions. The themes show the diversity of possible work with PE and that online PE may be a future worthwhile direction. Sarkhel et al. (117) described clinical practice guidelines for PE in different psychiatric disorders. They argue that PE remains a simple and cost-effective treatment modality that empowers patients and family members with knowledge about the illness, coping, and management. Despite the specific contributions of each model and the superior benefits of more intensive psychotherapeutic interventions compared with PE alone, these models may be difficult to implement in current practice as they require trained therapists. Facilitating access to online PE is interesting, and more research is needed in this domain. However, these studies have limitations, and more data are needed for persons with BD. There are currently different types of results with these types of interventions. For example, Yu et al. (118) conducted a systematic review to assess internet-based psychoeducation programs for caregivers of persons with dementia and showed the effects of the intervention on self-efficacy, anxiety, burden, and QoL remained inconclusive. By comparison, Tao et al. (119) conducted a systematic review of internet-based and mobile-based cognitive behavioral therapy for chronic disorders and showed that it was an effective intervention for comprehensive symptom management among people with major chronic medical conditions.

RCT studies are considered the most rigorous method for measuring the effectiveness of a new intervention or treatment. This designed is widely used for research in PE. However, Chakrabarti et al. (120) mentioned a gap of current best-evidence knowledge in the research on adjunctive PE for treatment of rapid-cycling BD. More evidence is also needed on the relative effectiveness of PE versus imagery focused CBT (121).

As an adjunct strategy to pharmacotherapy in treating BD, psychosocial interventions play an essential role in preventing new episodes, treating depressive episodes, reducing the frequency of new mood episodes and length of hospital stay, improving social functioning, and enhancing medication adherence.

PE focuses on illness awareness, adherence enhancement, detection of early warning signs, substance misuse avoidance, and lifestyle regularity (27). Combining PE and other interventions, such as CTB or Mindfulness, enables one to address more specifically. Combining PE and CBT can enhance symptom management and medication adherence, for example, or integrating PE and Mindfulness can heighten an acceptance attitude towards the illness and life difficulties.

For example, psychosocial interventions such as PE or IPSRT have proven effective. It can be hypothesized that combining interventions that impact treatment can produce a cumulative effect on the outcome, which relative effectiveness may be difficult to measure.

## 6 Conclusion

To conclude, the efficacy of psychoeducation in the treatment of BD is documented and is also used in combination with other interventions. Moreover, research on psychoeducation programs based totally or partially on digital models are sufficiently present in current literature to appears as a theme in this review. However, this type of intervention still lacks sufficient evidence.

## Author contributions

VL: Conceptualization, Data curation, Formal analysis, Methodology, Writing – original draft. SF: Data curation, Formal analysis, Writing – review & editing. HR-L: Conceptualization, Methodology, Supervision, Writing – review & editing.
