# Supplementary material for: Current practices of psychoeducation interventions with persons with bipolar disorders: a literature review
Source: Front Psychiatry. 2024 Jan 5;14:1320654. doi: 10.3389/fpsyt.2023.1320654 (PMC10797008; doi:10.3389/fpsyt.2023.1320654)
Supplement: Supplementary file 1 [file Table_1.docx]

Supplementary Table 1. Summary of articles included (continued)

| **Study** | **Population** | **No. of patients** | **Groups** | **Treatment** | **PE:lenght and frequency** | **Follow-up** | **Results** |
| --- | --- | --- | --- | --- | --- | --- | --- |
| **MODEL of COLOM and VIETA (continued)** |  |  |  |  |  |  |  |
| Scott et al. (2009) | BD I or II, ambulatory pat. in remission ≥ 6 months | EG : 60  GC : 60 | PE vs non-specific sessions | EG : TAU+ Group PE (8-12 pat.)  GC : TAU+ non specific group sessions | EG : 21 sessions x 90 min  GC : 20 weekly sessions | 5 years | EG :  -Twice as many outpatient appointments but estimated average cost of emergency consultations significantly lower  -More likely to opt for self-funded psychotherapy after completing PE  -More medication use  -Notably lower hospital costs |
| Javadpour et al. (2013) | BD, ambulatory ambulatory patients, in remission with recurrent episodes | EG : 54  GC : 54 | PE vs TAU | EG : individual PE + 18 x monthly telephone follow-up care  GC : TAU | EG : 8 weekly sessions x 50 min | 2 years | EG: (at 8 months)  -Improved medication adherence  -Improved QoL  -Fewer hospitalizations and relapse |
| Faria et al. (2014) | BD II, young ambulatory pat. (18-29 years) | EG : 32  GC : 29 | PE vs TAU | EG : PE  GC : TAU | EG : 6 sessions x 60 min. | Not specified | Depressive symptom remission in both groups  No significant reduction of manic symptoms in both groups  No difference in regulation of biological rhythms between the 2 groups |
| De Azevedo Cardoso et al. (2014) | BD II, young ambulatory pat. (18-29 years) | EG : 32  GC : 29 | PE vs TAU | EG : individual PE  GC : TAU | 6 sessions x 60 min. | 6 months | Improved QoL in both group except for the general health and physical pain items (post-intervention). Results maintained at 6 months, except for the physical health component (no significant difference) |
| De Azevedo Cardoso et al. (2015) | BD II, young ambulatory pat. (18-29 years) | EG : 32  GC : 29 | PE vs TAU | EG : PE individual setting  GC : TAU | 6 sessions x 60 min. | 12 months | No significant difference between the groups on symptoms (depressive, manic and anxiety) and on the regulation of biological rhythms at each follow-up |
| Gumus et al. (2015) | BD I or II, euthymic ≥3 months. | EG : 41  GC : 41 | PE vs TAU | EG : individual PE, adapted from Colom and Vieta, Miklowitz and Goldstein  GC : TAU | 4 weekly sessions x 60 min, | 12 months | EG :  -Fewer relapse and hospitalizations (no significant difference between the groups) |

Supplementary Table 1. Summary of articles included (continued)

| **Study** | **Population** | **No. of patients** | **Groups** | **Treatment** | **PE:lenght and frequency** | **Follow-up** | **Results** |
| --- | --- | --- | --- | --- | --- | --- | --- |
| **MODELof COLOM and VIETA (continued)** |  |  |  |  |  |  |  |
| Kallestad et al. (2016) | BD I or II, hardly no exclusion criteria | EG : 42  GC : 43 | Group PE vs individual PE | EG : Group PE +/- Motivational Interviewing (ME) (Miller et Rollnick) 8-12 pat.  GC : Individual PE based on Otto et al. (2003) manual | EG : 10 weekly sessions x 90min followed by 8 booster-sessions over the next two years,or three sessions of individual PE.  GC : 3 sessions x 60 min | 8 years follow-up including 27 months of intervention | -longer time to first admission over 27 months for EG  -trend maintained at 8 years (due to other clinical criteria, not to PE)  -reduced use of hospitalization (over the 8 year of follow-up) |
| Morriss et al. (2016) | BD I or II, at risk of relapse, euthymic ≥ 4 weeks | EG : 153  GC : 151 | Structured PE groups vs unstructured PE groups | EG : structured PE (Colom and Vieta, contextualized to UK practice (CANMAT, Yatham et al., 2013)  GC : unstructured PE (PARADES : peer support), 10-18 pat. | 21 weekly sessions x 120 min (26 weeks). | 96 weeks | EG : better participation rate  No significant difference between the 2 groups on the delay of the first relapse  PE : increased efficacy (delayed 1st relapse) when fewer past thymic episodes |
| Rahmani et al. (2016) | BD I, women, recruited at the hospital, euthymic during the study. | EG : 38  GC : 38 | PE vs TAU | EG: Group PE  GC: TAU | 10 bi-weekly session x 90 min | 5 weeks | EG : better medication adherence |
| Delle Chiaie et al. (2013) | BD, euthymic ≥ 5 months | EG : 9  GC : 11 | PE vs TAU | EG: Group PE  GC: Open discussion groups | 21 weekly sessions (unspecified lenght) | 5 months | No difference between the 2 groups (pre-post) on symptoms or medication adherence  EG: standardization of CAR |
| Wiener et al. (2017) | BD (18-29 y.o.) | EG : 32  GC : 29 | PE vs TAU | EG : PE  GC : TAU | 6 sessions x 60 min | Not specified | For the 2 groups: decrease in depressive symptoms, no difference on (hypo)manic symptoms (pre-post)  EG: increase in serum GDNF |

Supplementary Table 1. Summary of articles included (continued)

| **Study** | **Population** | **No. of patients** | **Groups** | **Treatment** | **PE:lenght and frequency** | **Follow-up** | **Results** |
| --- | --- | --- | --- | --- | --- | --- | --- |
| **MODELof COLOM and VIETA (continued)** |  |  |  |  |  |  |  |
| Bauer et al. (2006a, 2006b) | BD I or II, ambulatory pat. Symptomatic at inclusion, with co-morbidities | EG : 166  GC : 164 | PE vs TAU | EG : PE  GC : TAU | 5 weekly sessions x 60 min (phase 1) + weekly sessions (60 min) (2-17 months) (phase 2). | 3 years | EG :  -Reduction in the nb of weeks with a thymic episode (manic>depressive)  -Increased satisfaction with treatment  -No effect on average symptoms (manic and depressive)  -No difference on medication adherence |
| Bauer et al. (2009) | BD I ou II, ambulatory patients, symptomatic at inclusion, numerous comorbidities | EG : 166  GC : 164 | PE vs TAU | EG : PE  GC : TAU | 5 weekly sessions x 60 min (phase 1) + weekly sessions 60 min (2-17 months) (phase 2). | 3 years | EG : higher rates of guideline-concordant antimanic treatment than TAU over the entire follow-up period |

Supplementary Table 1. Summary of articles included (continued)

| **Study** | **Population** | **No. of patients** | **Groups** | **Treatment** | **PE:lenght and frequency** | **Follow-up** | **Results** |
| --- | --- | --- | --- | --- | --- | --- | --- |
| **MODEL of BAUER ET McBRIDE (suite)** |  |  |  |  |  |  |  |
| Sajatovic et al. (2009) | BD I or II, ambulatory pat. Euthymic or not | EG : 84  GC : 80 | PE vs TAU | EG : PE (6-8 pat.)  GC : TAU | EG: 6 weekly sessions (phase 1) + optional montly sessions (Phase 2) | 12 months | EG:  -41.49% of participation (=4-6 sessions of phase 1)  -No significative difference between the 2 groups regarding medication adherence, symptoms, and functioning |
| Parikh et al. (2012) | BD I or II, ambulatory patients, in remission ≥ 1 months | EG1 : 109  EG2 : 95 | PE vs CBT | EG1 : PE  EG2 : inidividual CBT | PE : 6 sessions x 90 min  CBT : 20 sessions x 50 min | 18 months | No difference between the 2 groups on relaspe, symptoms, and medication adherence  PE : lower cost per participant (180$ vs 1200$) |
| Parikh et al. (2013) | BD I or II, ambulatory patients, in remission ≥ 1 months | Total : 119  (unspecified group details) | PE vs CBT | EG1: PE  EG2: individual CBT | PE : 6 sessions x 90 min  CBT : 20 sessions x 50 min | 18 months | No difference between the 2 groups on improvement on symptom burden reduction  Equal improvement between the 2 groups on stimulus reduction and problem-focused coping styles  No change in acceptance of help-seeking behavior in both groups.  CBT : denial and blame reduction |
| So et al. (2021) | BDI or II, outpatients. | EG : 38  CG : 26 | PE vs waitlist control | EG : PE (phase 1 of LGP) – 5 to 7 pat.  CG : waitlist control (standard care/TAU followed by the same LGP intervention) | PE : 6 weekly sessions x 90 min | 6 months | Following LGP, improvements in :  - knowledge about illness and level of anxiety  - moment-by-moment mood stability  - medication adherence  Changes in anxiety and mood stability were significantly greater following LGP than CG. |
| **CBT** |  |  |  |  |  |  |  |
| Zaretsky et al. (2008) | BD I or II, ambulatory pat. euthymic in partial remission | EG1 : 39  EG2 : 40 | PE vs PE+CBT | EG1 : PE  EG2 : PE+ CBT  (individual PE and CBT are tape-recorded and based on Basco and Rush (1996) manual) | PE : 7 sessions (EG1)  +/- 13 weekly sessions of CBT (EG2)  (session duration=unspecified) | 12 months | EG1 : (PE): no increase in antidepressant dose  EG2 : (PE+CBT) : fewer days with depression over 1 year (50%)  No difference in hospitalization rate, medication compliance, psychosocial functioning, use of mental health care facilities |

| Miklowitz et al. (2007a) | BD I or II, ambulatory patients with depression | EG : 163 (IPSRT : 62, CBT : 75, FFT : 26)  CG : 130 | 3 types of intensive psychotherapy vs PE | EG : Intensive psychotherapy (IPSRT, CBT, FFT)  CG: Collaborative care (CC) = brief PE (individual sessions) | Weekly or bi-weekly Intensive Psychotherapy (up to 30 sessions in 9 months)  CC : PE 3 x 50 min in 6 sem. | 12 months | EG: (3 types of intensive psychotherapy)  -Higher remission rate at the end of follow-up  -Shorter time to return to remission  ->clinically stable throughout follow-up  -No significant difference between the 3 types of psychotherapy |
| --- | --- | --- | --- | --- | --- | --- | --- |

Supplementary Table 1. Summary of articles included (continued)

| **Study** | **Population** | **No. of patients** | **Groups** | **Treatment** | **PE:lenght and frequency** | **Follow-up** | **Results** |
| --- | --- | --- | --- | --- | --- | --- | --- |
| **CBT** (**continued)** |  |  |  |  |  |  |  |
| Miklowitz et al. (2007b) | BD I or II, ambulatory patients with depression | EG : 84 (IPSRT : 33, CBT : 38, FFT : 13)  GC : 68 | 3 types of intensive psychotherapy vs PE | EG : Intensive psychotherapy (IPSRT, CBT, FFT)  CG : Collaborative care (CC) = brief PE (individual sessions) | Weekly or bi-weekly intensive psychotherapy (up to 30 sessions in 9 months)  CC : PE 3 x 50 min in 6 weeks | 9 months | EG: (3 types of intensive psychotherapy)  -better global functioning  -better relational functioning  -better life satisfaction  -No difference in occupation and leisure |
| González Isasi et al. (2010) | BD I or II >2 years, euthymic but with severity criteria or unfavourable evolution despite adequate care | EG : 20  GC : 20 | Combined treatment vs TAU | EG : medication + PE + CBT  GC : medication | 20 group session x 90 min. | 12 months | EG :  -Fewer hospitalizations (at 12 months)  -Less depression and anxiety (at 6 and 12 months)  -Fewer (hypo)manic symptoms and lower scores on the misadjustment scale  -Reduced mania, depression, anxiety and misadjustment scores over time |
| González Isasi et al. (2014) | BD I or II >2 years, euthymic but with severity criteria or unfavourable evolution despite adequate care | EG : 20  GC : 20 | Combined treatment vs TAU | EG : medication + PE + CBT  GC : medication | 20 group session x 90 min. | 5 years | EG :  -less hospitalization events (at 12-months)  -lower depression and anxiety (at 6, 12 months and 5-years)  -Significant differences emerged in mania and misadjustment at 6, 12 and 5 years.  -less persistent affective symptoms and/or difficulties in social-occupational functioning after 5-year follow-up. |
| **DBT, MINDFULNESS and YOGA** |  |  |  |  |  |  |  |
| Van Dijk et al. (2013) | BD I or II, euthymic pat., or with depression or with hypomania | EG : 13  GC : 13 | Combined treatment vs waiting list | EG : TCD and mindfulness techniques + PE  GC : waiting list | EG : 12 group session x 90 min | 12 weeks | EG :  -Depressive symptoms reduction  -Increased awareness, diminished anxiety and better control of emotional states  -Fewer emergency room visits/admissions related to mental health within 6 months of combined treatment |
| Ravindran et al. (2020) | Uni or bipolar disorder, outpatients | EG 1 : 53  EG 2 : 19 | Yoga vs PE | EG 1 : manualized Breathing Focused Yoga (BFY), posture and guided meditation.  EG 2 : unspecific PE | EG 1 : sessions twice a week x 90 min  EG 2 : sessions twice a week x 90 min | 8 weeks | -no significant difference in MADRS ratings between intervention groups but significant decline in depressive symptoms for both groups.  -improvements in self-rated depressive symptoms and well-being for the 2 EG. |
| De Dios et al. (2021) | BP-outpatients with subthreshold depressive symptoms | EG1 : 34  EG2 : 40  CG : 10 | EG1 : TAU + PE  EG2 : TAU + MBCT  CG : TAU | EG1: PE (Colom and Vieta)  EG2  MBCT (developed by Kabat-Zinn)  CG : TAU | EG1 : 8x 2h weekly sessions  EG2 : 8 weekly sessions of 90 min | 6 months | -depressive symptoms improved in the 3 arms between baseline and 8 weeks and between baseline and 6 months  - no significant differences between groups were found at 6 months. |
| Valls et al. (2021) | BD I or II, euthymic or with subthreshold symptoms | EG : 47  CG: 47 | integrative approach + TAU vs TAU | EG : PE + session for family members only + mindfulness  training + cognitive and functional enhancement (10-14 pat.)  GC : pharmacological treatment | EG : 12 weekly group sessions x 90 min | 3 months | EG :  -Significant group × time interaction in favor of EG, improving the functional outcome.  -Significant effects in some domains of the FAST (cognitive domain, leisure time)  -significant group × time interaction in Hamilton Depression Rating Scale |
| **FFT** |  |  |  |  |  |  |  |
| Rea et al. (2003) | BD, recently hospitalized patients (for manic episode) | EG : 28  GC : 25 | FFT vs PE | EG : FFT (patient and family)  GC : individual PE | EG : FFT (21 sessions x 60 min)  GC : PE (21 sessions x 30 min)  12 weekly, 6 bi-weekly, 3 monthly sessions | 1 year treatment and 1 year follow-up | Active treatment phase (1 year) : no difference in the probability of relapse  During follow-up (2 years): no difference in medication adherence  EG:  -Fewer re-hospitalizations  -Fewer thymic relapse |

Supplementary Table 1. Summary of articles included (continued)

| **Study** | **Population** | **No. of patients** | **Groups** | **Treatment** | **PE:lenght and frequency** | **Follow-up** | **Results** |
| --- | --- | --- | --- | --- | --- | --- | --- |
| **FFT (continued)** |  |  |  |  |  |  |  |
| Miklowitz et al. (2000) | BDI, pat. recuited in hospital or outpatient settings | EG : 31  GC : 70 | Full FFT vs TAU + abbreviated FFT | EG : Full FFT(patient and family)  GC : TAU + 2 abbreviated sessions of FFT | EG : 21 sessions x 60 min, weekly (3months), then bi-monthly (3months), then monthly (3 months) | 12 months | EG :  -Fewer relapses  -Longer time to relapse  -Better improvement in depressive symptoms. No difference in manic symptoms. |
| Miklowitz et al. (2003) | BD, pat. recruited in hospital and outpatient settings, shortly after an accute episode | EG : 31  GC : 70 | FFT vs TAU (+ 2 FFT session and crisis sessions when needed) | EG : Full FFT (patient and family)  GC : TAU (+ 2 sessions of FFT and crisis sessions when needed) | EG : 21 sessions x 60 min, weekly (3months), then bi-monthly (3months), then monthly (3 months) | 2 years | EG :  -Fewer relapses  -Longer time to relapse  -Enhanced improvement on thymic symptoms  -Better medication adherence |
| Miklowitz et al. (2007a) | BD I or II, ambulatory patients with depression | EG : 163 (IPSRT : 62, CBT : 75, FFT : 26)  GC : 130 | 3 types of intensive psychotherapy vs PE | EG. Intensive psychotherapy (IPSRT, CBT, FFT)  CG : Collaborative care (CC) = brief PE (individual sessions) | Weekly or bi-weekly Intensive Psychotherapy (up to 30 sessions in 9 months)  CC : PE 3 x 50 min in 6 weeks | 12 months | EG: (3 intensive psychotherapy)  -Higher remission rate at the end of follow-up  -Shorter time to return to remission  -More stable clinically throughout the follow-up period  No significant difference in outcomes between the 3 types of intensive psychotherapy |
| Miklowitz et al. (2007b) | BD I or II, ambulatory patients with depression | EG : 84 (IPSRT : 33, CBT : 38, FFT : 13)  GC : 68 | 3 types of intensive psychotherapy vs PE | EG. Intensive psychotherapy (IPSRT, CBT, FFT)  CG: Collaborative care (CC) = brief PE (individual sessions) | Weekly or bi-weekly Intensive Psychotherapy (up to 30 sessions in 9 months)  CC : PE 3 x 50 min in 6 weeks | 9 months | EG: (3 intensive psychotherapy)  -Better overall functioning  -Better relational functioning  -Better life satisfaction  No difference on occupational and leisure functioning |
| **UNSPECIFIC PE INCLUDING FAMILY** |  |  |  |  |  |  |  |
| Clarkin et al. (1998) | BD, hospitalized or ambulatory pat., euthymic or not | EG : 18  GC : 15 | Couple PE vs TAU | EG : Couple PE sessions  GC : TAU | Couple PE (25 sessions : 10 weekly, 15 bimonthly) | 11 months | EG :  -Better global functioning  -Better pharmacologic compliance  No difference on symptoms |

Supplementary Table 1. Summary of articles included (continued)

| **Study** | **Population** | **No. of patients** | **Groups** | **Treatment** | **PE:lenght and frequency** | **Follow-up** | **Results** |
| --- | --- | --- | --- | --- | --- | --- | --- |
| **UNSPECIFIC PE INCLUDING FAMILY (continued)** |  |  |  |  |  |  |  |
| D’Souza et al. (2010) | BD, ambulatory patients, euthymic (≤ 1 months relapse) | EG : 27  GC : 31 | Groups of couple PE vs TAU | EG : Groups of couple PE  GC : TAU | 12 weekly sessions x 90 minutes | 60 weeks | EG :  -Fewer relapses  -Longer time to relapse  -Less (hypo)manic symptoms  -Better pharmacological compliance  No difference on depressive symptoms |
| **IPSRT** |  |  |  |  |  |  |  |
| Frank et al. (2005) | BD I or manic schizoaffective disorder symptomatic, with ≥ 3 past episodes | IPSRT/IPSRT : 22  ICM/ICM : 19  IPSRT/ICM : 27  ICM/IPSRT : 25 | 2 phases study: (acute and maintenance)  4 groups :  IPSRT (acute)/IPSRT (maintenance)  -ICM (acute)/ICM (acute)  -IPSRT (acute)/ICM (maintenance)  -ICM (acute)/IPSRT (maintenance) | -IPSRT  -ICM (Intensive Clinical Management) : « classic » PE model | IPSRT : 45 to 55 min  ICM : 20 to 25 min  Weekly sessions up to clinical stabilization, then every other week or week (during 12 weeks), then 1x/month in maintenance up to the end of the follow-up | 2 years | -Same time for thymic stability  -IPSRT (acute phase): longer time to thymic relapse  -IPSRT groups : improved regularity of social rhythms at the end of the acute phase of treatment  -Ability to improve regularity of social rhythms in acute phase correlated with reduced likelihood of relapse in maintenance phase |
| Frank et al. (2008) | BD I or manic schizoaffective disorder symptomatic, with ≥ 3 past episodes | IPSRT/IPSRT : 22  ICM/ICM : 19  IPSRT/ICM : 27  ICM/IPSRT : 25 | 2 phases study: (acute and maintenance)  4 groups :  IPSRT (acute)/IPSRT (maintenance)  -ICM (acute)/ICM (acute)  -IPSRT (acute)/ICM (maintenance)  -ICM (acute)/IPSRT (maintenance) | -IPSRT  -ICM (Intensive Clinical Mangement) : « classic » PE  model | IPSRT : 45 to 55 min  ICM : 20 to 25 min  Weekly sessions up to clinical stabilization, then every other week or week (during 12 weeks), then 1x/month in maintenance up to the end of the follow-up | 2 years | -Faster improvement in occupational functioning for the IPSRT vs ICM groups (mainly during acute treatment phase)  -No difference at 2 year follow-up |

Supplementary Table 1. Summary of articles included (continued)

| **Study** | **Population** | **No. of patients** | **Groups** | **Treatment** | **PE:lenght and frequency** | **Follow-up** | **Results** |
| --- | --- | --- | --- | --- | --- | --- | --- |
| **IPSRT (continued)** |  |  |  |  |  |  |  |
| **FUNCTIONAL REMEDIATION** |  |  |  |  |  |  |  |
| Torrent et al. (2013) | BD I or II, ambulatory patients in remission ≥ 3 months, moderate to severe degree of functional impairment | RF : 77  PE : 82  TAU : 80 | RF vs  PE vs TAU | EG1 : FR with tasks in session and at home  EG2 : PE base on Colom and Vieta model  TAU : medication | RF : 21 weekly sessions x 90 min  PE : 21 weekly sessions x 90 min | 6 months | EG (FR):  -Significant improvement in functioning compared to TAU  -No significant difference on functioning compared to PE  No significant difference on neurocognitive measures |
| Solé et al. (2015) | BD I or II, ambulatory patients in remission ≥ 3 months, moderate to severe degree of functional impairment | RF : 17  PE : 19  TAU : 17 | RF vs  PE vs TAU | EG1 : FR with tasks in session and at home  EG2 : PE based on Colom and Vieta model  TAU : medication | RF : 21 weekly sessions x 90 min  PE : 21 weekly sessions x 90 min | 6 months | EG (FR):  -Improved functioning (between T0 and at 6 months), suggesting an interaction between the program received and the time (pre-post), for FR compared to the 2 other groups  -Interaction between the program received and time on subclinical depressive symptoms, for FR (significant decrease) compared to PE.  -No significant difference in the evolution of subclinical manic symptoms. |
| Sanchez-Moreno et al. (2017) | BD I ou II, ambulatory patients with sub-syndromic symptoms, moderate to severe degree of functional impairment | RF : 33  PE : 37  TAU : 29 | RF vs  PE vs TAU | EG1 : FR with tasks in session and at home  EG2 : PE base on Colom and Vieta model  TAU : medication | RF : 21 weekly sessions x 90 min  PE : 21 weekly sessions x 90 min | 12 months | EG (FR):  -Significant time x group interaction in favor of the FR group on improvement of psychosocial functioning (compared to the 2 other groups)  No significant change on thymic symptoms in any group |

Supplementary Table 1. Summary of articles included (continued)

| **Study** | **Population** | **No. of patients** | **Groups** | **Treatment** | **PE:lenght and frequency** | **Follow-up** | **Results** |
| --- | --- | --- | --- | --- | --- | --- | --- |
| **FUNCTIONAL REMEDIATION (continued)** |  |  |  |  |  |  |  |
| Bonnin et al. (2016a) | BD I or II, ambulatory patients in remission ≥ 3 months, moderate to severe degree of functional impairment  + below average cognitive performance | RF : 56  PE : 69  TAU : 63 | RF vs  PE vs TAU | EG1 : FR with tasks in sessions and at home  EG2 : PE based on Colom et Vieta model  TAU : medication | RF : 21 weekly sessions x 90 min  PE : 21 weekly sessions x 90 min | 6 months | EG: FR > TAU but no to PE on improvement in delayed free recall (verbal memory)  -Improved functioning (compared to the other groups) |
| Bonnin et al. (2016b) | BD I or II, ambulatory patients in remission ≥ 3 months, severe degree of functional impairment | RF : 54  PE : 60  TAU : 58 | RF vs  PE vs TAU | EG1 : FR with tasks in sessions and at home  EG2 : PE based on Colom et Vieta model  TAU : medication | RF : 21 weekly sessions x 90 min  PE : 21 weekly sessions x 90 min | 12 months | EG (FR) :  -Improved verbal memory at 1 year  > TAU and PE on global functioning improvement.  No difference between the groups on the other neuropsychological variables (executive scores, processing speed, working memory attention) |
| Sachs et al. (2020) | BI I or II, remitted patients. | PE : 19  TAU : 24 | PE (cognitive) vs TAU | PE: cognitive psychoeducational group therapy (CPEGT) + sessions with relatives.  TAU : regular group sessions (book about BD).  For both groups, booster sessions (at 6 and 9 months) | PE : 14 sessions x 90 min  TAU : no information. | 12 months | Reduction of the number of depressive episodes after CPEGT. Benefit in the domain of occupational life. |
| **COLLABORATIVE CARE /REHABILITATION PROGRAM** |  |  |  |  |  |  |  |
| Van der Voort et al. (2015) | BD I, II, or NS. ambulatory patients, stable | EG : 56  GC : 82 | Collaborative Care vs TAU | EG : Collaborative care= PE + problem solving groups  GC : TAU | EG : 6 sessions x 120 min. | 12 months | EG :  -Less months spent with depressive symptoms (at 6 and 12 months).  -Diminished severity of depressive symptoms (at 12 months).  -No effect on manic symptoms or medication adherence. |
| Dalum et al. (2018) | Schizophrenia (76.5%), BD (23.5%), clinically stable or not | EG : 99  GC : 99 | Combined PE vs TAU | EG : PE group as part of IMR program (education about the disease and pharmacological treatments, coping and social skills training) – 10 patients groups  GC : TAU | EG : 1 weekly session x 60 min during 9 months | 9 months | No significant difference between the 2 groups on disease self-management, recovery, hope and satisfaction of the participants. |
| Jensen et al. (2019) | Schizophrenia (76.5%), BD (23.5%), clinically stable or not | EG : 99  GC : 99 | Combined PE vs TAU | EG : PE group as part of IMR program (education about the disease and pharmacological treatments, coping and social skills training) – 10 patients groups  GC : TAU | EG : 1 weekly session x 60 min during 9 months | 12 months | No significant difference between the 2 groups on global functioning, symptoms, number of hospitalizations, emergency visits, and use of ambulatory care. |

Supplementary Table 1. Summary of articles included (continued)

| **Study** | **Population** | **No. of patients** | **Groups** | **Treatment** | **PE:lenght and frequency** | **Follow-up** | **Results** |
| --- | --- | --- | --- | --- | --- | --- | --- |
| **COLLABORATIVE CARE / REHABILITATION PROGRAM**  (continued) |  |  |  |  |  |  |  |
| Shon et al. (2002) | Ambulatory schizophrenic patients, (55.26%), BD (28.95%), delusional disorder (15.79%) | EG : 18  GC : 20 | PE vs TAU | EG : PE group, not specific to BD. PE and rehabilitation program partly based on self-efficacy method (Bandura et al), video and telephone coaching  GC : TAU | EG : 12 sessions x 70 min | Not specified | EG :  -More improvement in self-efficacy  -Better medication adherence  -Lower scores of symptoms suggesting relapse |
| **PE AND UNSPECIFIED THEORICAL MODEL** |  |  |  |  |  |  |  |
| Eker et al. (2012) | BD, pat. in remission | EG : 35  GC : 36 | PE vs TAU + « informations » | EG : PE group, role playing, problem solving, (10-12 pat.)  GC : TAU + « informations » | EG : 6 sessions x 90-120 min (weekly) | 6 weeks. | EG: improved medication adherence |
| Çuhadar et al. (2014) | BD, ambulatory patients | EG : 24  GC : 23 | PE vs TAU | EG : PE standard + internal stigma component  GC : TAU | EG : 7 weekly sessions x 90 min | 7 weeks. | PE :  -Reduction on overall internalized stigma, as well as on the subscales (alienation, stereotype endorsement, perceived discrimination, and social withdrawal) |
| Luciano et al. (2021) | Ambulatory patients  BD (43.3%), schizophrenia or other psychotic disorder (29.9%), major depression (26.9%) | EG : 206  GC : 195 | Psychosocial group intervention vs PE | EG : PE + motivational interview + CBT + moderate physical activity, 5-10 pat.  GC : PE (unspecified theorical model), 5-10 pat. | EG : sessions every 7-10 days, including 20 min session of moderate physical activity  GC : 5 weekly sessions | 6 months | EG:  -Significant reduction in BMI and body weight  -Mean reduction in waist circumference |
| **PE FOR PATIENTS WITH SPECIFIC NEEDS** |  |  |  |  |  |  |  |
| Harvey et al. (2015) | BD I with inter-episode insomnia | CBT : 30  PE : 28 | CBT Vs PE | CBT: specific for sleep disorder  PE: not specific to sleep. Less than 10 minutes dedicated to sleep hygiene rules | 8 sessions x 50 à 60 min for both groups | 6 months | CBT group:  -Fewer days spent in an acute episode  -Less (hypo)manic relapses  -Lower (slightly) relapse rate  -Higher rate of insomnia remission (post treatment, but not at 6 months) |
| Sajatovic et al. (2017) | Depression (48%), schizophrenia (25%), BD (28%) + type 2 diabetes | EG : 100  GC : 100 | PE vs TAU | EG: LGP derived PE groups, problem identification, goal setting, behavioral modeling + PE focused on diabetes (6-10 patients)  GC : TAU | EG: 12 weekly sessions + 48 sem. of telephone follow-up | 60 weeks. | EG:  -Greater improvement in depressive symptoms, clinical global impression, and global functioning.  No difference for general health, psychological symptomatology (BPRS), and mean HbA1c levels |

Supplementary Table 1. Summary of articles included (continued)

| **Study** | **Population** | **No. of patients** | **Groups** | **Treatment** | **PE:lenght and frequency** | **Follow-up** | **Results** |
| --- | --- | --- | --- | --- | --- | --- | --- |
| **ONLINE PE** |  |  |  |  |  |  |  |
| Smith et al.  (2011) | BD I or II, ambulatory patients, in remission ≥ 3 months | EG : 24  GC : 26 | PE+TAU vs TAU | EG : online PE (mix between Colom and Vieta,model and LGP)  GC : TAU | PE : 8 sessions every 2 weeks for 4 months. | 10 months | No significant difference between the groups on QoL (except for the psychological subsection)  No difference on psychosocial functioning, insight, thymic symptomatology, relapses and use of care. |
| Proudfoot et al. (2012) | BD I or II | EG 1 : 139  EG 2 : 134  GC : 134 | Online PE vs online PE + peer support vs GC | EG1: online PE  EG2: online PE + email peer-support  GC: email links to sites with information about BD | PE : 8 weekly sessions x 30 à 40 min | 6 months | Increased perception of control, decreased perception of stigma, diminished anxiety and depression for all groups.  No significant difference between the 3 groups, except on depressive symptoms and level of functional impairment at 6 months (between EG1 and EG2)  Stronger adherence to the program if peer support is provided |
| Barnes et al. (2015) | BD I or II, euthymic pat. or not | EG : 113  GC : 120 | Online PE vs general information | EG: online PE +/- CBT sessions (optional) + mood self-assessment with feedback  CG: links to sites related to healthy lifestyle | EG et CG : 8 weekly sessions, followed by 2 sessions every 2 weeks, followed by 2 monthly sessions x 30-45 min (total : 20)  EG : 10 optional CBT sessions | 12 months | No significant difference between the groups on time to relapse. |

Supplementary Table 1. Summary of articles included (continued)

| **Study** | **Population** | **No. of patients** | **Groups** | **Treatment** | **PE:lenght and frequency** | **Follow-up** | **Results** |
| --- | --- | --- | --- | --- | --- | --- | --- |
| **ONLINE PE**  **(continued)** |  |  |  |  |  |  |  |
| Gliddon et al. (2019) | BDI I, II or NS | EG1 : 102  EG2 : 100  GC : 102 | PE vs PE+CBT vs TAU | EG1: Forum discussion + PE  EG2: Forum discussion+ PE + interactive CBT tools  GC : Forum discussion (peer-support) + TAU | For both EG: 5 sessions 2x/ week + 4 reminder sessions at 3, 6, 9 et 12 months | 12 months | EG1: improvement on depression scores compared to CG  Both EG: improvement on MADRS sub-items (apparent sadness, described sadness, internal tension, lassitude, inability to feel, pessimistic thoughts) compared to CG  No difference on mania scores. No difference on QoL, medication adherence and mental health functioning |
| Depp et al. (2015) | BD I ou II, ambulatory patients, euthymic or with few symptoms | EG : 51  GC : 53 | PE + interactive follow-up with smartphone vs  PE + mood journalling | -Individual PE for both groups, adapted form LGP  -Smartphone intervention : association of thymic state with self-efficacy strategies | PE: 4 x 1h (both groups)  Smartphone follow-up or mood diary (10 weeks) | 6 months | High satisfaction rate for both groups. No difference on mania scores and functional impairment.  EG: greater decrease in depressive symptoms (at 6 and 12 weeks but not at 24 weeks) |
| Moore et al. (2015) | BD I or II + documented HIV infection | EG : 25  GC : 25 | PE + SMS vs  PE | EG 1: individual PE (with therapist) on medication adherence + treatment reminder messages + mood assessment  EG 2: individual PE (with therapist) + daily mood assessment  => PE adapted from Colom and Vieta manual | PE : sessions x 30 min | 30 days | Medication adherence: high and comparable for both groups. No difference on the moment of taking psychotropic drugs.  EG1: antiretrovirals taken closer to schedules time than CG |
| Bilderbeck et al. (2016) | BDI or II, ≥ 16 years, pat. euthymic | EG : 60  GC : 61 | PE with therapist vs self-administered PE | EG1: PE manual+ individual PE with therapist + online mood self-assessment (email/SMS)  EG2: PE manual (self-management) + online mood self-assessment (email/SMS) | EG1: PE = 5 sessions x 50 min over 12 weeks | 12 months | No difference between groups on the evolution of depressive symptomatology, relapse rate, and hospitalization.  EG1: better knowledge of BD at 3months, correlated with a greater proportion of weeks of clinical stability (over 12 months) |
| Dupuis Maurin et al. (2020) | BD I, II or NS, pat. euthymic ≥ 3 months | EG : 20  GC : 21 | PE + serious game vs  PE | -PE based on Colom and Vieta manual for both groups  -Serious game: *BIPOLIFE* (an avatar suffering from BD. Goal: > 3 consecutive days of euthymia) | PE: 12 weekly sessions x 90 minutes | 4 months  (1 month using *BIPOLIFE*) | EG:  -Medical adherence lower at baseline (not at 1 and 4 months) compared to CG  -Absolute variation of the scores of the medication adherence scale and of the beliefs towards treatment scale higher in EG at 1 month (not at 4) compared to CG  No difference in the number of consultation (scheduled or emergency). Low rate of connection to the game despite a high satisfaction rate. |

Supplementary Table 1. Summary of articles included (continued)

| **Study** | **Population** | **No. of patients** | **Groups** | **Treatment** | **PE: lenght and frequency** | **Follow-up** | **Results** |
| --- | --- | --- | --- | --- | --- | --- | --- |
| **ONLINE PE**  **(continued)** |  |  |  |  |  |  |  |
| Petzold et al. (2019) | BD I or II, pat. in remission ≥ 2 months | EG : 39  GC : 34 | PE + structured computerized daily life self-observation vs  GC | EG: PE groups with computerized program for daily life structured self-observation (ChronoRecord). Reported data: once a day (5-10 patients).  CG: not-structured groups + not-structured self-observation | EG : PE = 6 weekly sessions x 90 min | 54 weeks | No difference in the number of thymic relapses. No difference in time to the first relapse.  No significant time x group interaction on: thymic symptoms, QoL, perceived involvement in care, self-efficacy expectations and locus of control (towards health) |
| Depp et al. (2019) | Schizophrenia, schizoaffective disorder (71.4%), BDI (28.6%). Ambulatory patients | EG *CBT2go*: 85  EG *SM*: 85  GC: 85 | CBT2go vs SM vs TAU | *CBT2go*: individual PE (CBT) and interactive self-observation with smartphone (CBT) + telephone follow-up  *SM*: General individual PE and interactive self-observation with smartphone (not CBT) + telephone follow-up  GC : TAU | PE 1 session (90 min) for both EG | 24 weeks | Both EG:  Improved symptomatology (0-24 weeks)  -*CBT2go* not different from *SM* on symptomatology.  *CBT2go* significant time x group interaction with better functioning scale scores 24 weeks (not for SM).  -*CBT2go* lower scores on pessimistic performance beliefs (not for SM). |

| Murray et al. (2021) | BD I, II or NOS, ≥ 10 episodes. | EG: 152  CG: 150 | ORBIT 2.0 vs PE | ORBIT 2.0: brief online mindfulness-based intervention (coach-supported program) + TAU  GC: online PE (coach-supported program) + TAU | EG and CG: 4 modules (1/week over 4 weeks) + 1 week to consolidate skill development with the online coach. | 6 months | EG was not superior to CG in improving QoL.  The platform was safe, both interventions were highly acceptable.  Suboptimal usage. |
| --- | --- | --- | --- | --- | --- | --- | --- |

Note: BD: bipolar disorder, pat: patients, PE: psychoeducation, EG: experimental group, CG: control group, min. minutes, weeks, ttt: treatment, TAU: treatment as usual, , QALY: quality-adjusted life year, CAR: cortisol awakening response, GDNF: glial cell line-derived neurotrophic factor, LGP: Life Goals Program, CBT: cognitive behavioral therapy, IPSRT: interpersonal and social rhythm therapy, FFT: family focused therapy, CC: collaborative care, DBT: behavioral dialectical therapy, RF: functional remediation, QoL : Quality of Life.
